# Decisions and Decisional Needs of Canadians From all Provinces and Territories During the COVID-19 Pandemic: Population-Based Cross-sectional Surveys

**DOI:** 10.2196/43652

**Published:** 2023-03-21

**Authors:** Dawn Stacey, Claire Ludwig, Patrick Archambault, Maureen Smith, Monica Taljaard, Meg Carley, Karine Plourde, Laura Boland, Amédé Gogovor, Ian Graham, Daniel Kobewka, Robert K D McLean, Michelle L A Nelson, Brandi Vanderspank-Wright, France Légaré

**Affiliations:** 1 School of Nursing Faculty of Health Sciences University of Ottawa Ottawa, ON Canada; 2 Centre for Implementation Research Ottawa Hospital Research Institute Ottawa, ON Canada; 3 Patient Partner Ottawa, ON Canada; 4 Department of Family Medicine and Emergency Medicine Université Laval Quebec City, QC Canada; 5 Centre de recherche intégrée pour un système apprenant en santé et services sociaux Université Laval Lévis, QC Canada; 6 VITAM - Centre de recherche en santé durable Université Laval Quebec City, QC Canada; 7 Cochrane Consumer Network Executive Ottawa, ON Canada; 8 School of Epidemiology and Public Health University of Ottawa Ottawa, ON Canada; 9 Department of Medicine University of Ottawa Ottawa, ON Canada; 10 General Internal Medicine The Ottawa Hospital Ottawa, ON Canada; 11 International Development Research Centre Ottawa, ON Canada; 12 Sinai Health System University of Toronto Toronto, ON Canada; 13 Dalla Lana School of Public Health University of Toronto Toronto, ON Canada

**Keywords:** health care decisions, decisional conflict, decision regret, shared decision-making, COVID-19, older adults, caregivers, parents, public health decision, health care, health outcome, pandemic preparedness, public health policy

## Abstract

**Background:**

Never before COVID-19 had Canadians faced making health-related decisions in a context of significant uncertainty. However, little is known about which type of decisions and the types of difficulties that they faced.

**Objective:**

We sought to identify the health-related decisions and decisional needs of Canadians.

**Methods:**

Our study was codesigned by researchers and knowledge users (eg, patients, clinicians). Informed by the CHERRIES (the Checklist for Reporting Results of Internet E-Surveys) reporting guideline, we conducted 2 online surveys of random samples drawn from the Leger consumer panel of 400,000 Canadians. Eligible participants were adults (≥18 years) who received or were receiving any health services in the past 12 months for themselves (adults) or for their child (parent) or senior with cognitive impairment (caregiver). We assessed decisions and decisional needs using questions informed by the Ottawa Decision Support Framework, including decisional conflict and decision regret using the Decision Conflict Scale (DCS) and the Decision Regret Scale (DRS), respectively. Descriptive statistics were conducted for adults who had decided for themselves or on behalf of someone else. Significant decisional conflict (SDC) was defined as a total DCS score of >37.5 out of 100, and significant decision regret was defined as a total DRS score of >25 out of 100.

**Results:**

From May 18 to June 4, 2021, 14,459 adults and 6542 parents/caregivers were invited to participate. The invitation view rate was 15.5% (2236/14,459) and 28.3% (1850/6542); participation rate, 69.3% (1549/2236) and 28.7% (531/1850); and completion rate, 97.3% (1507/1549) and 95.1% (505/531), respectively. The survey was completed by 1454 (97.3%) adults and 438 (95.1%) parents/caregivers in English (1598/1892, 84.5%) or French (294/1892, 15.5%). Respondents from all 10 Canadian provinces and the northern territories represented a range of ages, education levels, civil statuses, ethnicities, and annual household income. Of 1892 respondents, 541 (28.6%) self-identified as members of marginalized groups. The most frequent decisions were (adults vs parents/caregivers) as follows: COVID-19 vaccination (490/1454, 33.7%, vs 87/438, 19.9%), managing a health condition (253/1454, 17.4%, vs 47/438, 10.7%), other COVID-19 decisions (158/1454, 10.9%, vs 85/438, 19.4%), mental health care (128/1454, 8.8%, vs 27/438, 6.2%), and medication treatments (115/1454, 7.9%, vs 23/438, 5.3%). Caregivers also reported decisions about moving family members to/from nursing or retirement homes (48/438, 11.0%). Adults (323/1454, 22.2%) and parents/caregivers (95/438, 21.7%) had SDC. Factors making decisions difficult were worrying about choosing the wrong option (557/1454, 38.3%, vs 184/438, 42.0%), worrying about getting COVID-19 (506/1454, 34.8%, vs 173/438, 39.5%), public health restrictions (427/1454, 29.4%, vs 158/438, 36.1%), information overload (300/1454, 20.6%, vs 77/438, 17.6%), difficulty separating misinformation from scientific evidence (297/1454, 20.4%, vs 77/438, 17.6%), and difficulty discussing decisions with clinicians (224/1454, 15.4%, vs 51/438, 11.6%). For 1318 (90.6%) adults and 366 (83.6%) parents/caregivers who had decided, 353 (26.8%) and 125 (34.2%) had significant decision regret, respectively. In addition, 1028 (50%) respondents made their decision alone without considering the opinions of clinicians.

**Conclusions:**

During COVID-19, Canadians who responded to the survey faced several new health-related decisions. Many reported unmet decision-making needs, resulting in SDC and decision regret. Interventions can be designed to address their decisional needs and support patients facing new health-related decisions.

## Introduction

During the COVID-19 pandemic, never before had Canadians made health-related decisions in the context of so much uncertainty. Health care decisions were complex, with limited, rapidly changing evidence and evolving public health directives [1,2]. Concurrently, health services shifted from in-person to virtual delivery and emergency department visits increased after the first wave [3-5]. These circumstances are perfectly fit for shared decision-making, a process between patients and clinicians that relies on the best evidence available and what matters most to patients. However, due to unique challenges with communicating risk, lack of nonverbal communication, and less meaningful patient involvement experienced during the pandemic, it may have been possible that Canadians were inadequately involved in health-related decisions [2,6,7]. When patients are inadequately involved in health decisions, there are more harms from choosing ineffective options, health care system waste, poor patient experiences, more litigation, and higher inequities [8,9]. This is why identifying Canadians decision-making needs was of uttermost importance in order to be able to provide person-centered care, achieve improved health outcomes, and inform future pandemic preparedness.

Decisional needs are deficits that can adversely affect the quality of decisions [10]. A quality decision is informed with the best-available evidence and based on patients’ values for features and outcomes of options. A previous systematic review of 45 decisional needs assessment studies included 2 population surveys that identified 75 decisions (including 16 social decisions) and 43 clinical studies that focused on 29 specific health decisions [10]. Common decisional needs included decisional conflict, inadequate knowledge of the options (including benefits, harms), unclear values, and limited support and resources [10,11]. Decisional conflict refers to “uncertainty about a course of action when choice among competing options involves risk, regret, or challenge to personal life values” [12]. Those who experience more difficultly with decision-making are living with a serious or chronic illness, are immunocompromised, belong to linguistic minorities, have lower education, are passive in decision-making, or make a decision on behalf of someone else (parents and caregivers) [13-19]. None of the 45 studies from 7 countries were focused on decision-making needs during pandemics [10]. A recent cross-sectional study of 4905 Canadians aged 18-40 years reported that factors associated with vaccine hesitancy are negative attitudes toward vaccines in general; COVID-19 conspiracy theory beliefs; distrust of the government; and a low income, low education, or unemployment [20]. Canada was no exception, as little was known about its health-related decision-making experience during the COVID-19 pandemic. Therefore, we sought to determine the type of decisions and decisional needs of Canadians during the first year of the COVID-19 pandemic.

## Methods

### Study Design

Our team of researchers and knowledge users (patients, clinicians) conducted 2 population-based cross-sectional surveys using Leger’s consumer panel. Having patients and clinicians as equal partners on the team was done to ensure the study yielded relevant findings [21]. Patients were on the executive research team coleading the study (author CL who is immunocompromised), and author MS stimulated the research topic. Both were engaged in all study aspects. Together, we identified the research objectives, outcomes, procedures, and deliverables for the funded proposal. Next, they were involved in guiding the study. We used the CHERRIES (Checklist for Reporting Results of Internet E-Surveys) guideline [22].

### Setting

The survey was conducted in Canada during the third wave of COVID-19 [23]. During the first wave of COVID-19 in January-June 2020, 80% of the COVID-19–related deaths occurred in long-term-care homes, and governments issued stay-at-home orders and travel restrictions [24]. Masks became mandated for use in indoor spaces in July 2020. The second wave of COVID-19 started in November 2020 and the third wave in March 2021. When the hospitals became overwhelmed during the third wave, with a high impact on intensive care units, governments reissued a stay-at-home order [23]. COVID-19 vaccines were initially approved by Health Canada in December 2020, with priority for health care workers, Indigenous peoples, the elderly living in group settings, and others at higher risk (eg, people with cancer, with organ transplants, or undergoing dialysis). Vaccination gradually opened to adults in spring 2021 and to children (ages 5-12 years) in fall 2021 [25]. Although the AstraZeneca vaccine was approved for use on February 26, 2021, the rate of vaccine-induced immune thrombotic thrombocytopenia was of concern (1 in 60,000; April 2021). Governments continued to recommend the AstraZeneca vaccine until June 2021 because Canada was in the third wave of infections and there was insufficient supply of messenger RNA (mRNA) vaccines [23].

### Recruitment

We recruited 2 groups of participants: (1) adults aged 18 years or older who received or were receiving any health services in the past 12 months for themselves (labeled “adults”) and (2) adults aged 18 years or older who were responsible for children or seniors aged >65 years with cognitive impairment who received or were receiving any health services in the past 12 months (labeled “parents/caregivers”). Leger recruited participants through its consumer panel titled “Leger Opinion (LEO) Panel,” which has about 400,000 Canadians across 10 provinces and 3 territories. The panel includes data on age, gender, and region that can be used for sampling and quota management. At the onset, specific quotas by age, gender, and region were set based on the Canadian population data published by Statistics Canada [26]. Leger monitored throughout the recruitment phase, and sampling was adjusted to ensure the data collected were representative. For example, if a certain cohort was underrepresented, sampling was adjusted to recruit more respondents falling within that cohort. There was no weighting applied to the data after being collected. Respondents received a personalized email containing a unique URL link to the survey. The email invite said, “LEO wants to hear from you!” and it did not state the exact survey topic.

### Survey Instrument

Adapted from previous surveys in Canada [13,14,27], questions were based on the Ottawa Decision Support Framework [11]. Questions from instruments with good reliability and validity included the Decisional Conflict Scale (DCS) [12], the Decision Regret Scale (DRS) [28], and Strull’s roles in decision-making [13]. The survey enquired about health-related decisions adults and parents/caregivers faced during the past 12 months, factors influencing decision-making, trusted information sources, and sociodemographics according to the PROGRESS (place of residence, race/ethnicity/culture/language, occupation, gender/sex, religion, education, socioeconomic status, and social capita) framework [29,30]. To collect data on a broader range of participants’ characteristics that may stratify health opportunities and outcomes due to discrimination [29,30], we also asked respondents to self-identify if they had lived experience as a member of a marginalized group defined as disabled or caregiver of a person with a disability, gender diverse (eg, agender, nonbinary, transgender, cisgender), intersex, LGBTQ+ (lesbian, gay, bisexual, pansexual, transgender, queer, two-spirited, questioning), Indigenous, racialized (eg, person of color), neurodivergent (eg, attention deficit hyperactivity disorder [ADHD], autism, dyslexia), or a marginalized group not listed. Respondents were initially asked to list all the decisions made in the past year and then pick 1 difficult decision for the remaining questions. A difficult decision was defined as having more than 1 option and no clear best option. The online survey, available in English or French, screened eligibility and included 32 questions (Multimedia Appendix 1). Two random test questions were used to ensure respondents were not trying to speed through the survey, and the survey stopped for those who clicked a wrong response on both.

Respondents received 1 question per screen and pressed “continue” to proceed. They could not return to previous screens to change responses. To avoid missing responses, no questions could be skipped. Up to 3 reminders were sent to those who started but did not complete the survey. To avoid multiple entries, respondents were assigned a unique identifier linked to their LEO account; if they tried to complete the survey again, they received an error message.

The English and French versions were pretested by members of our team, including patient partners. On May 18, 2021, we field-tested the surveys with 139 adults and 26 parents/caregivers to monitor completion time (adults: ~10 minutes; parents/caregivers: ~9 minutes) and potential problems with surveys, including predefined skip logic. Given no changes were made to the surveys based on field testing, the collected data were retained.

### Sample Size

Based on our previous research [13,31,32], sample sizes of 1500 adults and 500 parents/caregivers were adequate to estimate proportions with significant decisional conflict (SDC) associated with decision delay. SDC is defined as a total score of >37.5 out of 100 on the DCS based on 253 studies [33] using a 2-sided 95% CI with a margin of error of +1.80% or +3.85% when the estimated proportions are 0.15 or 0.26, respectively [34,35]. This is the most severe cutoff; sensitivity analyses were performed with a more relaxed cutoff of 25, also sometimes seen in the literature (data not shown), but we privileged a more severe cutoff. This means it could have been showing more people with SDC. To account for an anticipated 10% response rate, target sample sizes of adults and parents/caregivers were 15,000 and 5000, respectively.

### Statistical Analysis

We classified respondents into adults who made a health-related decision for themselves (adults) or made a decision on behalf of a child or a senior with cognitive impairment (parents/caregivers). Next, we used descriptive statistics to identify decisions and described decisional needs using the Ottawa Decision Support Framework for each group (adults and parents/caregivers) [10]. We classified respondents as having SDC [36], and for those who had made the decision, we classified respondents as having significant decision regret based on a cutoff of >25 out of 100 on the DRS [37].

### Ethical Considerations

The University of Ottawa research ethics board approved our study (H-03-21-6752). Invitees provided study consent at the start of the online survey and only initiated the survey questions after clicking the link consenting to participate. Respondents voluntarily answered questions and were guaranteed confidentiality. Leger offered an incentive of 2000 points (equivalent to CA $1.60, or US $1.18) to complete the survey.

## Results

### Respondent Details

**Figure 1:**
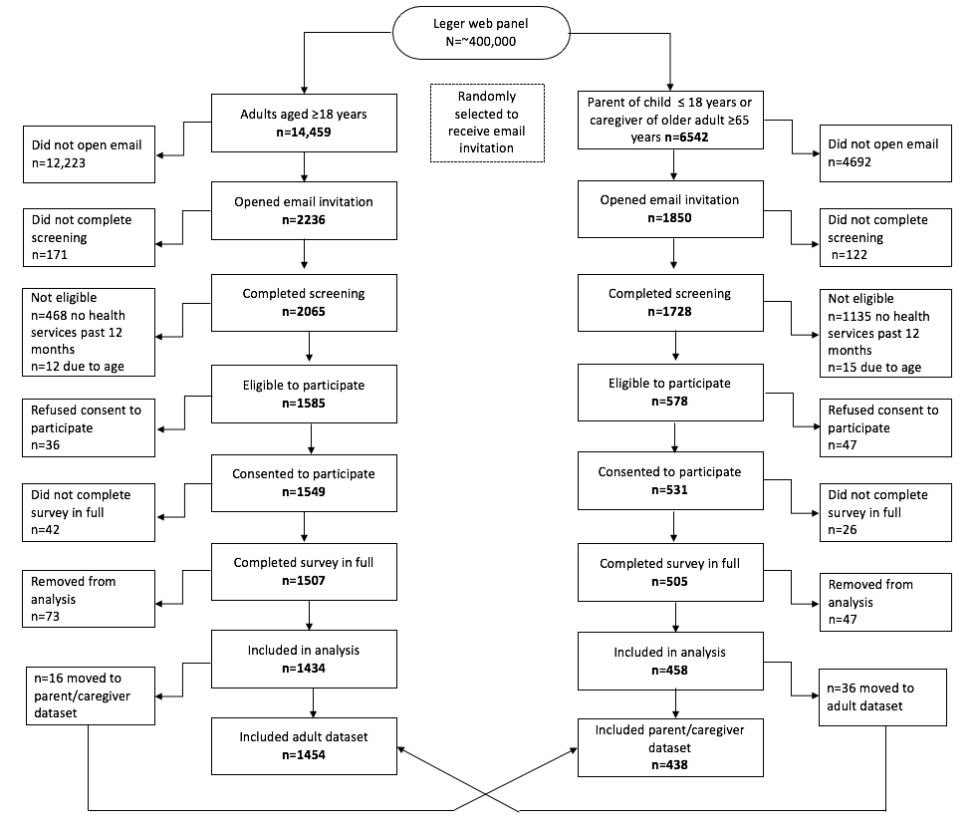
Cross-sectional survey recruitment of Canadians during the COVID-19 pandemic.

From May 18 to June 4, 2021, 14,459 adults and 6542 parents/caregivers were invited to participate (Figure 1). The view rate (unique survey visitors/unique survey invitees) was 15.5% (2236/14,459) and 28.3% (1850/6542), respectively. The participation rate (unique visitors who consented to participate/unique survey visitors) was 69.3% (1549/2236) and 28.7% (531/1850). The completion rate (users who completed the survey/users who consented to participate) was 97.3% (1507/1549) and 95.1% (505/531), respectively. Among those who completed the surveys, we removed respondents if they did not identify a difficult health decision. After reading open text describing options, 36 were moved to the adult database and 16 were moved to the parent/caregiver database.

There were 1454 adults and 438 parents/caregivers included in the analysis. The survey was completed in English (1598/1892, 84.5%) or French (294/1892, 15.5%). Respondents represented a range of ages, education levels, civil statuses, ethnicities, and annual household income (Table 1). Of 1892 respondents, 541 (28.6%) self-identified as members of marginalized groups.

**Table 1:** Demographic characteristics of Canadian cross-sectional survey respondents during the COVID-19 pandemic.

Variable			Adults (N=1454)		Parents/caregivers (N=438)
**Age (years), n (%)**					
	18-29	264 (18.2)		46 (10.5)	
	30-39	238 (16.4)		117 (26.7)	
	40-49	259 (17.8)		126 (28.8)	
	50-59	248 (17.1)		92 (21.0)	
	60-69	250 (17.2)		41 (9.4)	
	≥70	195 (13.4)		16 (3.7)	
**Provinces/territories, n (%)**					
	Ontario	558 (38.4)		165 (37.7)	
	Quebec	264 (18.2)		84 (19.2)	
	Prairie Provinces	269 (18.5)		87 (19.9)	
	British Columbia	241 (16.6)		69 (15.8)	
	Atlantic Provinces	115 (7.9)		30 (6.8)	
	Northern Territories	7 (0.5)		3 (0.7)	
**Geographical area, n (%)**					
	Urban	1269 (87.3)		393 (89.7)	
	Rural	185 (12.7)		45 (10.3)	
**Sex, n (%)**					
	Female	789 (54.3)		219 (50.0)	
	Male	664 (45.7)		218 (49.8)	
	Prefer not to say	1 (0.1)		1 (0.2)	
**Gender, n (%)**					
	Woman	781 (53.7)		219 (50.0)	
	Man	662 (45.5)		218 (49.8)	
	Other^a^ or prefer not to say	11 (0.8)		1 (0.2)	
**Language first learned and still understood^b^, n (%)**					
	English	1023 (70.4)		290 (66.2)	
	French	270 (18.6)		92 (21.0)	
	Mandarin or Cantonese	46 (3.2)		13 (3.0)	
	Other^c^	124 (8.5)		43 (9.8)	
**Highest level of education, n (%)**					
	High school or less	303 (20.9)		67 (15.3)	
	Certificate/diploma below bachelor’s level	528 (36.3)		157 (35.8)	
	Bachelor’s degree	372 (25.6)		136 (31.1)	
	University degree above bachelor’s level	249 (17.1)		75 (17.1)	
	Prefer not to say	2 (0.1)		3 (0.7)	
**Cultural/ethnic background^b,d^, n (%)**					
	White	1149 (79.0)		314 (71.7)	
	Asian	206 (14.2)		84 (19.2)	
	North American Indigenous	53 (3.6)		17 (3.9)	
	Black	22 (1.5)		17 (3.9)	
	Latin American	20 (1.4)		10 (2.3)	
	European	25 (1.7)		4 (0.9)	
	Other	26 (1.8)		7 (1.6)	
	Prefer not to say	17 (1.2)		7 (1.6)	
**Lived experience as member of a marginalized group^b,e^, n (%)**					
	Disabled or caregiver of a person with a disability	142 (9.8)		68 (15.5)	
	LGBTQ+^f^	133 (9.1)		32 (7.3)	
	Gender diverse: eg, agender, nonbinary, trans-/cis-gender	26 (1.8)		8 (1.8)	
	Racialized as person of color	109 (7.5)		44 (10.0)	
	Neurodivergent: eg, ADHD^g^, autistic, dyslexic	60 (4.1)		13 (3.0)	
	Indigenous	29 (2.0)		13 (3.0)	
	Other	26 (1.8)		5 (1.1)	
	None of the above	1038 (71.4)		283 (64.6)	
	Prefer not to say	25 (1.7)		5 (1.1)	
**Civil status, n (%)**					
	Married or common law	838 (57.6)		321 (73.3)	
	Single, divorced, or separated	570 (39.2)		108 (24.7)	
	Widowed	38 (2.6)		7 (1.6)	
	Prefer not to say	8 (0.6)		2 (0.5)	
Number in household, mean (SD; range)			2.6 (1.3; 1-11)		3.3 (1.3; 1-8)
**Annual household income (CA $/US $)^h^, n (%)**					
	<50,000/<36761.75	325 (22.4)		56 (12.8)	
	50,000-99,999/36761.75-73522.76	554 (38.1)		214 (48.9)	
	≥100,000/≥73523.50	450 (30.9)		146 (33.3)	
	Prefer not to say	125 (8.6)		22 (5.0)	
Quality of life^i^, mean (SD)			4.8 out of 7 (1.4)		4.7 out of 7 (1.4)

^a^Examples of other genders specified included bisexual, demiboy, gender fluid, transgender, and 2-spirited.

^b^Respondents sometimes specified more than 1 response option.

^c^Other languages included Arabic, Armenian, Bengali, Croatian, Czech, Danish, Dutch, Filipino, Finnish, Fukien, Friulan, German, Greek, Haitian Creole, Hakka, Hindi, Irish, Italian, Japanese, Kannada, Konkani, Korean, Laotian, Latvian, Macedonian, Malayalam, Mongolian, Norwegian, Pashto, Polish, Portuguese, Punjabi, Romanian, Russian, Serbian, Slovak, Spanish, Tagalog, Taiwanese, Tamil, Teochew, Ukrainian, Urdu, Vietnamese, Yoruba, and Zulu.

^d^Other cultural/ethnic backgrounds included Acadian, African, Canadian, Caribbean, Fijian, Guyanese, Jewish, mixed, and West Indian.

^e^Other reasons respondents indicated having lived experience as a member of a marginalized group included intersex, their gender (eg, “a woman”), occupation, social capital, socioeconomic status, religion, philosophical or political beliefs, physical or mental health condition, and not specified.

^f^LGBTQ+: lesbian, gay, bisexual, pansexual, transgender, queer, two-spirited, questioning.

^g^ADHD: attention deficit hyperactivity disorder.

^h^CA $1=US $0.74.

^i^Overall quality of life was measured on a 7-point scale: from 1 (life is very distressing, and it is difficult to imagine how it could get much worse) to 7 (life is great, and it is difficult to imagine how it could get much better); 4, life is so-so, neither good nor bad.

### Decisions

Health-related decisions, in order of frequency, were about COVID-19 vaccination, managing a health condition, social COVID-19 decisions (masking, limiting contacts), mental health care or addiction treatment, medication, surgery, pain management, health care for COVID-19 (testing, seeking care for symptoms), stay at home or move to an assisted care facility (eg, nursing home), pregnancy, birth control, staying safe at home (eg, adapt or retrofit home), and end-of-life care (Table 2). Decisions were similar when respondents were asked to pick a single decision described as more difficult, with the exception of health care for COVID-19, which a few rated as difficult. Other single decisions for caregivers were about moving a family member to/from a retirement or nursing home and stopping them from driving a car. Decisions (adults vs parents/caregivers) were within the past month (318/1318, 24.1%, vs 70/366, 19.1%), 1-6 months (524/1318, 39.8%, vs 134/366, 36.6%), 6-12 months (307/1318, 23.3%, vs 97/366, 26.5%), or no response (169/1318, 12.8%, vs 65/366, 17.8%).

**Table 2:** Health-related decisions of Canadian cross-sectional survey respondents during the COVID-19 pandemic.

Decisions			Adults, n/N (%)					Parents/caregivers, n/N (%)		
		All decisions		One difficult decision^a^		All decisions			One difficult decision^a^	
COVID-19 vaccination			1180/1454 (81.2)		490/1454 (33.7)		235/438 (53.7)			87/438 (19.9)
COVID-19 social decisions: eg, masking, limiting contacts			975/1454 (67.1)		158/1454 (10.9)		243/438 (55.5)			85/438 (19.4)
**Health care for COVID-19**			350/1454 (24.1)		39/1454 (2.7)		114/438 (26.0)			29/438 (6.6)
	Testing	N/A^b^		27/39 (69.2)		N/A			18/29 (62.1)	
	Seeking care of COVID-19 symptoms	N/A		9/39 (23.1)		N/A			2/29 (6.9)	
	Participating in clinical trials	N/A		1/39 (2.6)		N/A			0	
	Not specified	N/A		2/39 (5.1)		N/A			9/29 (31.0)	
**COVID-19: delaying medical treatment/visits**			N/A		120/1454 (8.3)		N/A			29 (6.6)
	Health condition	N/A		87/120 (72.5)		N/A			15/29 (51.7)	
	Surgery	N/A		20/120 (16.7)		N/A			9/29 (31.0)	
	Mental health care	N/A		13/120 (10.8)		N/A			3/29 (10.3)	
	Chemotherapy	N/A		0		N/A			2/29 (6.9)	
Pregnancy and childbirth			N/A		23/1454 (1.6)		N/A			N/A
Move temporarily from nursing or retirement home			13/1454 (0.9)		1/1454 (0.1)		30/438 (6.8)			6/438 (1.4)
Options to stay safe at home or move to have proper support and assistance			N/A		N/A		N/A			8/438 (1.8)
**Managing a health condition**			641/1454 (44.1)		166/1454 (11.4)		159/438 (36.3)			30/438 (6.8)
	Delaying medical treatment/visits	N/A		92/166 (55.4)		N/A			7/30 (23.3)	
	Having tests	N/A		27/166 (16.3)		N/A			7/30 (23.3)	
	New treatments	N/A		14/166 (8.4)		N/A			4/30 (13.3)	
	Dental visit	N/A		26/166 (15.7)		N/A			1/30 (3.3)	
	Admission to hospital	N/A		1/166 (0.6)		N/A			1/30 (3.3)	
	Other	N/A		2/166 (1.2)		N/A			2/30 (6.7)	
	Not specified	N/A		4/166 (2.4)		N/A			8/30 (26.7)	
**Mental health care decisions**										
	Treatment	326/1454 (22.4)		115/1454 (7.9)		78/438 (17.8)			24/438 (5.5)	
	Addiction or overdose	20/1454 (1.4)		2/1454 (0.1)		26/438 (5.9)			2/438 (0.5)	
**Medication decisions**			477/1454 (32.8)		115/1454 (7.9)		138/438 (31.5)			23/438 (5.3)
	Antibiotics	N/A		1/115 (0.9)		N/A			1/23 (4.3)	
	Lower cholesterol	N/A		6/115 (5.2)		N/A			0	
	Control blood sugar	N/A		3/115 (2.6)		N/A			1/23 (4.3)	
	Prevent heart burn	N/A		4/115 (3.5)		N/A			0	
	Sleeping pills	N/A		10/115 (8.7)		N/A			1/23 (4.3)	
	Other	N/A		10/115 (8.7)		N/A			3/23 (13.0)	
	Not specified	N/A		81/115 (70.4)		N/A			17/23 (73.9)	
Pain management		327/1454 (22.5)		88/1454 (6.1)		83/438 (18.9)			15/438 (3.4)	
**Surgery decisions**			153/1454 (10.5)		69/1454 (4.7)		54/438 (12.3)			12/438 (2.7%)
	Delay surgery	N/A		56/69 (81.2)		N/A			10/12 (83.3)	
	Joint replacement	N/A		7/69 (10.1)		N/A			0	
	Prostate cancer	N/A		2/69 (2.9)		N/A			0	
	Back surgery	N/A		4/69 (5.8)		N/A			0	
	Breast cancer	N/A		0		N/A			1/12 (8.3)	
	Not specified	N/A		0		N/A			1/12 (8.3)	
**Pregnancy or childbirth**			101/1454 (6.9)		24/1454 (1.7)		7/438 (1.6)			N/A
	Planning a pregnancy	N/A		17/24 (70.8)		N/A			N/A	
	Prenatal testing	N/A		1/24 (4.2)		N/A			N/A	
	Childbirth type or setting	N/A		3/24 (12.5)		N/A			N/A	
	Methods of feeding	N/A		2/24 (8.3)		N/A			N/A	
	Unplanned pregnancy	N/A		1/24 (4.2)		N/A			N/A	
Birth control			162/1454 (11.1)		34/1454 (2.3)		4/438 (0.9)			N/A
**End of life**			14/1454 (1.0)		4/1454 (0.3)		28/438 (6.4)			13/438 (3.0)
	Mechanical ventilator	N/A		1/4 (25.0)		N/A			3/13 (23.1)	
	Palliative care	N/A		0		N/A			2/13 (15.4)	
	Medical Assistance in Dying (MAiD)	N/A		2/4 (50.0)		N/A			1/13 (7.7)	
	Advanced care planning	N/A		0		N/A			1/13 (7.7)	
	Location of care	N/A		1/4 (25.0)		N/A			6/13 (46.2)	
**Other**										
	Stay home or move (eg, nursing home)	6/1454 (0.4)		N/A		92/438 (21.0)			42/438 (9.6)	
	Best option to stay safe at home	6/1454 (0.4)		N/A		103/438 (23.5)			20/438 (4.6)	
	Stop driving car	5/1454 (0.3)		2/1454 (0.1)		52/438 (11.9)			13/438 (3.0)	
	Participating in clinical trials	42/1454 (2.9)		1/1454 (0.1)		1/438 (0.2)			N/A	
	Smoking cessation	N/A		1/1454 (0.1)		N/A			N/A	
	Being more active/eating healthy	N/A		1/1454 (0.1)		N/A			N/A	
	Moving to another location	N/A		1/1454 (0.1)		N/A			N/A	

^a^Identify 1 specific difficult health care decision that you faced or are facing.

^b^N/A: not applicable.

### Decisional Needs

**Figure 2:**
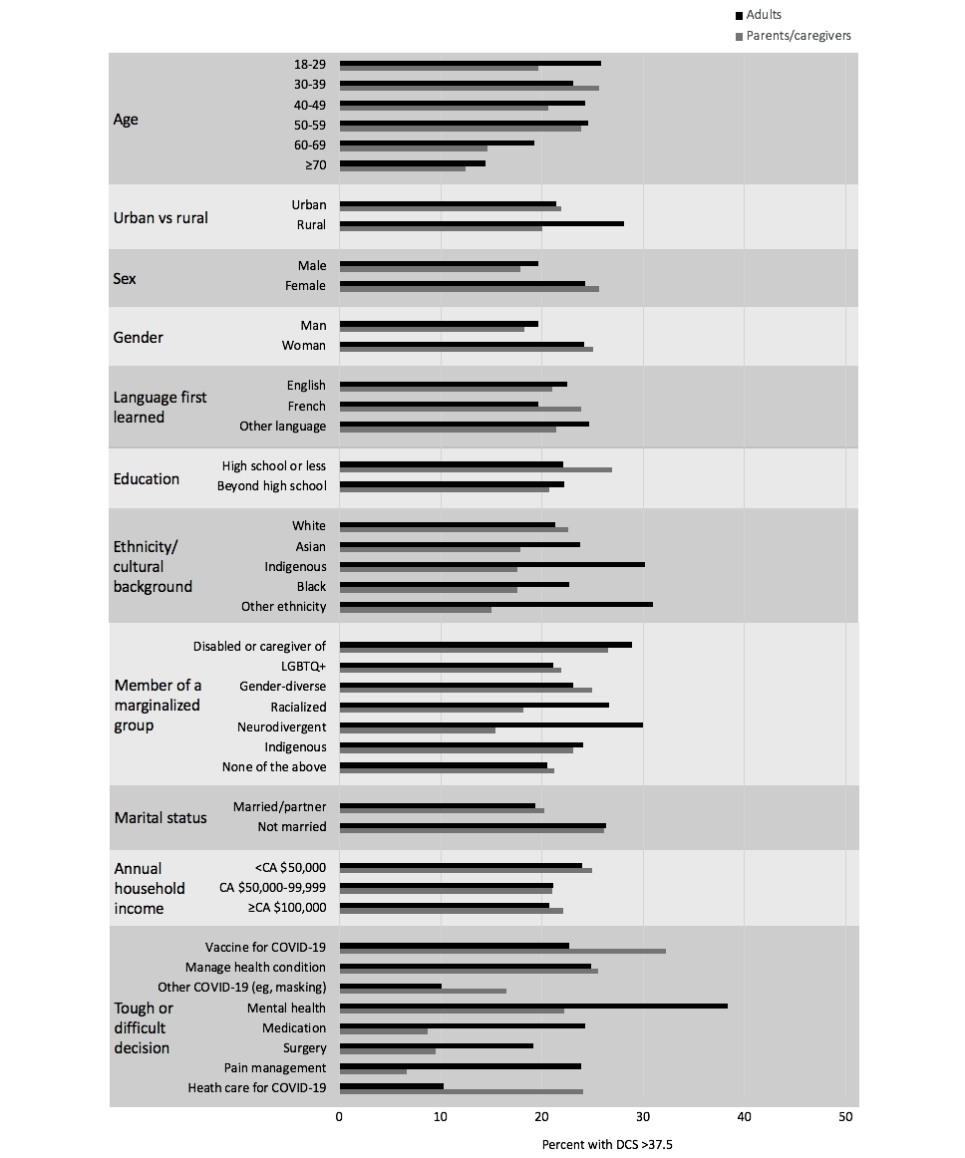
Proportion of Canadian cross-sectional survey respondents with clinical SDC>37.5 out of 100 during the COVID-19 pandemic. CA $1=US $0.74. DCS: Decisional Conflict Scale; LGBTQ+: lesbian, gay, bisexual, pansexual, transgender, queer, two-spirited, questioning; SDC: significant decisional conflict.

Of 1454 adults and 438 parents/caregivers, 323 (22.2%) and 95 (21.7%), respectively, had SDC (Tables 3 and 4). Decisions, in order of frequency, for adults with SDC were mental health care, managing a health condition, taking medications, pain management, and COVID-19 vaccination (Figure 2). Decisions for parents/caregivers with SDC were COVID-19 vaccination, managing a health condition, health care for COVID-19, and mental health care.

**Table 3:** Decisional needs and factors influencing decision-making of Canadian cross-sectional survey respondents during the COVID-19 pandemic (DCS^a^).

Variables			Adults (N=1454)		Parents/caregivers (N=438)
DCS score>37.5 out of 100			323 (22.2)		95 (21.7)
**Total decisional conflict (DCS)^b^**					
	Mean (SD)	25.5 (17.3)		26.2 (16.9)	
	Median (Q1, Q3)^c^	25.0 (12.5, 36.3)		25.0 (14.1, 35.9)	
**DCS uncertain subscale**					
	Mean (SD)	31.8 (23.6)		32.2 (21.9)	
	Median (Q1, Q3)	25.0 (16.7, 50.0)		29.2 (16.7, 50.0)	
**DCS uninformed subscale**					
	Mean (SD)	23.5 (19.2)		23.0 (17.4)	
	Median (Q1, Q3)	25.0 (8.3, 33.3)		25.0 (8.3, 33.3)	
**DCS unclear values subscale**					
	Mean (SD)	23.4 (19.6)		24.1 (19.8)	
	Median (Q1, Q3)	25.0 (0.0, 33.3)		25.0 (8.3, 33.3)	
**DCS unsupported subscale**					
	Mean (SD)	26.9 (20.8)		28.8 (21.4)	
	Median (Q1, Q3)	25.0 (8.3, 41.7)		25.0 (16.7, 41.7)	
**DCS ineffective decision subscale**					
	Mean (SD)	22.9 (19.5)		23.7 (18.7)	
	Median (Q1, Q3)	25.0 (6.3, 31.3)		25.0 (6.3, 31.3)	
Worried about choosing the wrong option, n (%)			557 (38.3)		184 (42.0)
Worried about getting COVID-19, n (%)			506 (34.8)		173 (39.5)
Public health restrictions due to COVID-19, n (%)			427 (29.4)		158 (36.1)
Overloaded with information, n (%)			300 (20.6)		77 (17.6)
Difficulty separating misinformation from scientific evidence, n (%)			297 (20.4)		77 (17.6)
Difficulty discussing the decision with important others (eg, spouse, family, friends), n (%)			192 (13.2)		89 (20.3)
Difficulty discussing the decision with clinicians, n (%)			224 (15.4)		51 (11.6)
No or limited access to information on the decision or options, n (%)			173 (11.9)		62 (14.2)
Difficulty in believing scientific evidence, n (%)			158 (10.9)		44 (10.0)
No skills or ability for making this type of decision, n (%)			91 (6.3)		40 (9.1)
Other (eg, unable to see the doctor in person to manage the health condition, side effects of the COVID-19 vaccine), n (%)			195 (13.4)		32 (7.3)
Considered the costs related to the options, n (%)			356 (24.5)		134 (30.6)

^a^DCS: Decisional Conflict Scale.

^b^Respondents sometimes specified more than 1 response option.

^c^Q1: quartile 1; Q3: quartile 3.

**Table 4:** Decisional needs and factors influencing decision-making of Canadian cross-sectional survey respondents during the COVID-19 pandemic (DRS^a^).

Variables		Adults who made a decision (N=1318)	Parents/caregivers who made a decision (N=366)
**Decisional regret^b^**			
	Mean (SD)	18.8 (18.2)	21.3 (18.4)
	Median (Q1, Q3)^c^	20.0 (0.0, 35.0)	25.0 (5.0, 40.0)
No decisional regret=0, n (%)		367 (27.8)	89 (24.3)
Low decisional regret=1 to ≤25, n (%)		598 (45.4)	152 (41.5)
Decisional regret>25, n (%)		353 (26.8)	125 (34.2)
Decision made alone, n (%)		843 (58.0)	185 (50.5)
Preferred option chosen, n (%)		996 (75.6)	289 (79.0)

^a^DRS: Decision Regret Scale.

^b^Values were standardized out of 100.

^c^Q1: quartile 1; Q3: quartile 3.

Respondents experiencing SDC were more likely to feel uninformed (mean 44, SD 18.6, vs mean 18, SD 14.3, out of 100), have unclear values (mean 45, SD 20.3, vs mean 18, SD 14.6), feel they made an ineffective decision (mean 48, SD 16.1, vs mean 16, SD 13.3), and have decision regret (mean 41, SD 17.5, vs mean 16, SD 15.7) compared to those not experiencing SDC. In addition to feeling worried about choosing the wrong option (193/418, 46.2%), respondents experiencing SDC identified the following factors as making the decisions more difficult: public health restrictions due to COVID-19 (133/418, 31.8%), difficulty separating misinformation from scientific evidence (113/418, 27.0%), information overload (107/418, 25.6%), and no or limited access to information on the decision (97/418, 23.2%); see Table 3 for all respondents. Adults identified difficulty discussing the decision with clinicians (85/323, 26.3%). Parents/caregivers identified difficulty discussing the decision with significant others (eg, family, friends; 22/95, 23.2%).

Of 1318 (90.6%) adults and 366 (83.6%) parents/caregivers who made a decision, 353 (26.8%) and 125 (34.2%), respectively, had decision regret (Table 4). Those facing mental health care decisions had higher decision regret (mean 32.0%, SD 46.8%). Adults (322/1318, 24.4%) and parents/caregivers (77/366, 21.0%) reported not getting their preferred option.

### Role in Decision-Making

Most respondents indicated an active role in making the decision either alone (768/1318, 58.3%, adults vs 181/366, 49.5%, parents/caregivers) or after considering the opinion of their clinician (348/1318, 26.4%, adults vs 105/366, 28.7%, parents/caregivers). Few decided together with their clinician (148/1318, 11.2%, adults vs 63/366, 17.2%, parents/caregivers) or deferred to their clinician (54/1318, 4.1%, adults vs 17/366, 4.6%, parents/caregivers). If asked to make the decision again, most preferred an active role (1067/1318, 81.0%, adults vs 264/366, 72.1%, parents/caregivers) or together with their clinician (218/1318, 16.5%, adults vs 83/366, 22.7%, parents/caregivers).

### Trusted Information Sources

Respondents indicated that trustworthy information sources were health professionals (1190/1454, 81.8%, adults vs 323/438, 73.7%, parents/caregivers), Health Canada (931/1454, 64.0%, adults vs 273/438, 62.3%, parents/caregivers), and provincial health departments (755/1454, 51.9%, adults vs 211/438, 48.2%, parents/caregivers). Fewer respondents trusted information from specific health organizations (583/1454, 40.1%, adults vs 167/438, 38.1%, parents/caregivers), consumer/patient organizations (265/1454, 18.2%, adults vs 79/438, 18.0%, parents/caregivers), companies that produce health information (199/1454, 13.7%, adults vs 49/438, 11.2%, parents/caregivers), or health insurance companies (99/1454, 6.8%, adults vs 43/438, 9.8%, parents/caregivers). Those experiencing SDC and decision regret were less trusting of these sources (Table 5).

**Table 5:** Trusted information sources for Canadian cross-sectional survey respondents during the COVID-19 pandemic.

Variable	Adults					Parents/caregivers			
	DCS^a^≤37.5 (n=1131)	DCS>37.5 (n=323)	DRS^b^≤25 (n=965)	DRS>25 (n=353)	DCS≤37.5 (n=343)		DCS>37.5 (n=95)	DRS≤25 (n=241)	DRS>25 (n=125)
Health professional	943 (83.4)	247 (76.5)	828 (85.8)	264 (74.8)	261 (76.1)		62 (65.3)	211 (87.6)	76 (60.8)
Health Canada	763 (67.5)	168 (52.0)	676 (70.1)	186 (52.7)	226 (65.9)		47 (49.5)	166 (68.9)	73 (58.4)
Provincial health departments	622 (55.0)	133 (41.2)	551 (57.1)	152 (43.1)	177 (51.6)		34 (35.8)	141 (58.5)	51 (40.8)
Specific health organizations	463 (40.9)	120 (37.2)	400 (41.5)	130 (36.8)	135 (39.4)		32 (33.7)	104 (43.2)	43 (34.4)
Consumer/patient associations	204 (18.0)	61 (18.9)	170 (17.6)	66 (18.7)	64 (18.7)		15 (15.8)	41 (17.0)	21 (16.8)
Companies that produce health information	150 (13.3)	49 (15.2)	123 (12.7)	57 (16.1)	39 (11.4)		10 (10.5)	27 (11.2)	16 (12.8)
Health insurance companies	81 (7.2)	18 (5.6)	66 (6.8)	23 (6.5)	36 (10.5)		7 (7.4)	20 (8.3)	13 (10.4)

^a^DCS: Decisional Conflict Scale.

^b^DRS: Decision Regret Scale.

## Discussion

### Principal Findings

In the first year of the pandemic, the most frequent decisions identified by Canadians were about COVID-19 vaccination, managing a health condition, social COVID-19 decisions, mental health care, medication treatments, and caregiver decisions about moving family members to or from residential facilities. One in five respondents had SDC, and a third reported decision regret. Factors making decisions more difficult were public health restrictions due to COVID-19, information overload, difficulty separating misinformation from scientific evidence, and difficulty discussing decisions with clinicians. Demographics of and the types of decisions made by respondents indicating iniquities above a level of 30% differences were ethnicity and mental health, respectively. The most trusted information sources were health care professionals and governmental sources. Our results led to the following observations:

- Respondents described 2 broad types of decisions, COVID-19–specific decisions and “routine” health-related decisions influenced by the pandemic or pandemic-related changes to health care services (eg, virtual care). When respondents were asked to focus on 1 difficult decision, some COVID-19–related decisions were selected less often and these decisions were more likely to have been influenced by mandatory public health regulations (eg, mask wearing in public indoor spaces, COVID-19 testing before an exposed child goes to school) [1,6]. However, the common decision about COVID-19 vaccination, selected by those experiencing SDC, would have been influenced by emerging scientific evidence and changing recommendations from Canadian public health officials [38]. COVID-19 vaccination became available to Canadian adults 5 months prior to the survey, with priority for frontline health care workers, the elderly in residential care, and Indigenous peoples [39]. In addition, 2 months prior to the survey, 4 vaccines were approved in Canada and public health officials recommended vaccination for adults and encouraged pregnant or breastfeeding persons to make shared decisions with their clinicians [38]. Decisions were being made when COVID-19 misinformation was spreading rapidly on social media, health care services were mostly virtual, and risk communication was also challenged with reports that the AstraZeneca vaccine caused rare blood clots [2-4,40-43]. In fact, misinformation also influenced uptake of the influenza vaccine in the United Kingdom [43].
- Many respondents reported decisional conflict and were worried about choosing the wrong option, a known manifestation of decisional conflict [33,44]. The 22.2% who reported SDC in our survey using the 16-item measure of decisional conflict were less than the 59% who reported being unsure about what to choose (1-item measure of decisional conflict) in the 1999 population-based study of 635 Canadians [17]. Another study conducted in March 2020 prior to the COVID-19 pandemic reported that 14.6% of 460 Canadian adults aged 65 years and older receiving home care services had SDC and common difficult decisions were about housing [14]. The highest proportion of respondents experiencing SDC in our study were making mental health care decisions. This was not surprising, given mental health has been impacted the most during COVID-19 [45,46]. Respondents reporting SDC highlighted unmet decisional needs requiring targeted support to address underlying modifiable factors [11]. Effective interventions for addressing decisional conflict are patient decision aids [11,47]. During our survey, 3 publicly available decision aids specific to COVID-19 were available (eg, moving a loved one from a retirement or nursing home, vaccination for persons who were pregnant or breastfeeding) [48-50]. However, not all information is accessible. Racialized and Indigenous Canadians described that the barriers to understanding COVID-19 public health information were the use of unfamiliar medical terminology, limited to English or French, and requiring technology to access the information (eg, the internet, television) [51]. Hence, our findings indicate the importance of monitoring and better supporting those experiencing SDC with plain-language interventions designed to ensure accessibility for all Canadians.
- For respondents who made a decision, a third reported decision regret. Decision regret was previously reported in 54% of 932 Canadian caregivers of seniors receiving health care services in their home in February 2020 [27]. Those with decision regret in both studies had SDC as well as a mismatch between their preferred option and the decision made. Decision regret is associated with lower satisfaction, lower quality of life, and decisional conflict [28]. Although regret can be managed by using interventions to ensure a realistic understanding of options and expectations of outcomes [11,52], our findings showed that respondents who were experiencing decision regret had lower levels of trust in the information provided by national, provincial, or local organizations. In addition, their decisions were influenced by information overload, difficulty separating misinformation from scientific evidence, and difficulty believing scientific evidence. Another survey reported that vaccine-hesitant Canadians aged 18-40 years were influenced by conspiracy theories and a distrust of governments [20]. Furthermore, protests against public health regulations indicate high levels of distrust of COVID-19 information [53]. This highlights the need to find ways to correct misinformation and help people make informed decisions [42,43]. More importantly, as we know that decision regret may lead to more litigation/complaints, we cannot completely exclude that this may be a mechanism by which there is an increasingly fractured society, with increasingly more Canadians feeling deceived by public officials, including public health officials.
- Our surveys found more patient-controlled decision-making compared to previous surveys [6,17]. We are unsure whether these findings are related specifically to the context of the COVID-19 pandemic or a change in Canadians’ roles. Half of the respondents made their decisions alone, but some preferred a more collaborative role with their clinicians. Our findings are higher than the 29% who decided alone in the 1999 Canadian survey [17] and different from a survey of 1061 Germans who preferred clinician-led decisions for hypothetical COVID-19 situations [6]. A few respondents (<6%) in our study had their clinicians make the decision for them or their clinicians make the decision after considering the respondents’ opinion. Another consideration is that most health care services were virtual during the pandemic [3,7] and likely impacted roles in decision-making.
- Lastly, we found demographics and types of health-related decisions showing iniquities among Canadians, mostly ethnicity and mental health. As our health care system is espousing the quintuple objective (better patient outcomes, better patient experiences, better efficiency, better health team well-being, and more equity [54,55]), this should be resonating in public health policies in terms of future interventions targeting the most vulnerable people in our society.

### Strengths and Limitations

The involvement of knowledge users on the study team was beneficial, and they guided all aspects of this study, including survey design, interpretation of findings, and drafting of results. No negative effects of involving knowledge users were encountered. Our view rate of 15.5% for adults and 28.3% for parents/caregivers was comparable to the typical rate for online surveys of 10%-20% [27,56-58]. In addition, the geographic distribution across provinces and territories was consistent with Canadian distribution [59].

With regard to limitations, our data may be subject to recall bias, given respondents were asked to identify a difficult decision made within the previous year. Second, it would have been easier to compare findings with the 1999 survey had we used “facing a complex health decision” as an eligibility criterion [17]. However, we were interested in all decisions (including those with any level of SDC or decision regret) for those who had interactions within the health care system. Compared to Canadian census data, fewer respondents had high school education or less (33.7% invited, 20.5% participated, 44.8% census) and were from the Canadian territories (0.2%-0.4% invited, 0.5% from Yukon participated, 0.1% census); see Multimedia Appendix 2. Finally, the question about costs influencing the decision preceded the question asking to list options, and some only listed options related to costs.

### Conclusion

Our survey of Canadians identified COVID-19–related decisions that emerged during the first year of the pandemic and how the pandemic influenced other health-related decisions. Many adults, parents, and caregivers had unmet decision-making needs, resulting in SDC and decision regret. Factors making decisions more difficult were public health restrictions due to COVID-19, information overload, difficulty separating misinformation from scientific evidence, and difficulty discussing decisions with clinicians. Most Canadians made the decisions on their own, with few sharing the decisions with others. Canadians need their decision-making needs recognized for providing person-centered care to achieve improved health outcomes and to inform future pandemic preparedness.

## Appendix

Multimedia Appendix 1 Supporting Canadians making health decisions: a decisional needs assessment during the COVID-19 pandemic – questionnaire.

Multimedia Appendix 2 Comparison of respondents to those invited and the Canadian census.

## Abbreviations

ADHD: attention deficit hyperactivity disorder
DCS: Decisional Conflict Scale
DRS: Decision Regret Scale
LEO: Leger Opinion
LGBTQ+: lesbian, gay, bisexual, pansexual, transgender, queer, two-spirited, questioning
SDC: significant decisional conflict

## Data Availability

The data sets used and analyzed during this study are available from the corresponding author upon reasonable request.
