# Supplementary material for: Decisions and Decisional Needs of Canadians From all Provinces and Territories During the COVID-19 Pandemic: Population-Based Cross-sectional Surveys
Source: JMIR Public Health Surveill. 2023 Mar 21;9:e43652. doi: 10.2196/43652 (PMC10131685; doi:10.2196/43652)
Supplement: Multimedia Appendix 1 [file publichealth_v9i1e43652_app1.docx]

**Appendix 1**

**SUPPORTING CANADIANS MAKING HEALTH DECISIONS: A DECISIONAL NEEDS ASSESSMENT DURING THE COVID-19 PANDEMIC – QUESTIONNAIRE**

**(ENGLISH VERSION)**

**SCREENER**

S.0 Would you prefer to complete the survey in English or French?

Préféreriez-vous répondre à ce questionnaire en anglais ou en français?

*Please select one answer only.*

*Veuillez sélectionner une seule réponse.*

| a. | 🔾 | English / Anglais | Go to S1 in English |
| --- | --- | --- | --- |
| b. | 🔾 | French / Français | Go to S1 in French |

S1. What is your date of birth? *Please enter in Day/Month/Year.*

**PN: PLEASE PROGRAM A CALENDAR**

**___________**

**PN: THANK AND TERMINATE IF LESS THAN 18 YEARS OLD**

S2**.** Have you received any health services during the last 12 months?

| YES | 1 |  |
| --- | --- | --- |
| NO | 2 | **THANK AND TERMINATE** |

S3**.** What is the date (month and year) during which you last received health services? *Please enter in Month/Year.*

**___________**

**PN: THANK AND TERMINATE IF MORE THAN 12 MONTHS AGO**

**PN: SHOW ON A SEPARATE SCREEN**

You are invited to participate in a survey intended to be completed by adults receiving or have received health services in the last year. It is designed to learn more about adults’ needs when decisions are being made about their health. The survey will take about 15 to 20 minutes to complete. This research is funded by the Canadian Institutes of Health Research. For more information about the research project please contact the Research Project Manager or visit the Research website: <https://decisionaid.ohri.ca>.

**MAIN QUESTIONNAIRE**

**SECTION A: SOCIO-DEMOGRAPHIC INFORMATION ON PARTICIPANTS [PN: DO NOT SHOW]**

**PN: SHOW ON A SEPARATE SCREEN**

To begin the survey, I would like to ask some background information about you. This information will be used to help us describe the group of people who filled out the survey and help us analyze the findings.

A1. In which province or territory do you live?

| Newfoundland and Labrador | 1 |
| --- | --- |
| Prince Edward Island | 2 |
| Nova Scotia | 3 |
| New Brunswick | 4 |
| Quebec | 5 |
| Ontario | 6 |
| Manitoba | 7 |
| Saskatchewan | 8 |
| Alberta | 9 |
| British-Colombia | 10 |
| Nunavut | 11 |
| Northwest Territories | 12 |
| Yukon | 13 |
| Other, please specify ______ | 96 |

A2. What was your sex at birth? Sex refers to sex assigned at birth.

| Male | 1 |
| --- | --- |
| Female | 2 |
| Prefer not to say | 99 |

A3. What is your gender? Refers to current gender which may be different from sex assigned at birth and may be different from what is indicated on legal documents.

| Male | 1 |
| --- | --- |
| Female | 2 |
| Other, please specify _____ | 96 |
| Prefer not to say | 99 |

A4. What is the language that you first learned at home in childhood and can still understand?

| French | 1 |
| --- | --- |
| English | 2 |
| An Aboriginal language | 3 |
| Spanish | 4 |
| Mandarin | 5 |
| Arabic | 6 |
| Others, please specify _____ | 96 |

A5. What is your highest level of education received?

| Less than high school diploma or its equivalent | 1 |
| --- | --- |
| High School diploma or equivalency certificate | 2 |
| College, CEGEP or other non-university certificate or diploma (other: trades certificates or diplomas) | 3 |
| University certificate or diploma below bachelor’s level | 4 |
| Bachelor’s degree (e.g., B.A., B.A. (Hons), B.Sc., LL.B.) | 5 |
| University certificate or diploma or degree above the bachelor’s level | 6 |
| Other, please specify _____ | 96 |
| Prefer not to say | 99 |

A6. What are the first 3 characters of your postal code?

__________ **[PN: ALLOW 3 DIGITS]**

A7. People living in Canada come from many different cultural and ethnic backgrounds. Are you?

| First Nations | 1 |
| --- | --- |
| Métis | 2 |
| Inuk | 3 |
| White | 4 |
| Black | 5 |
| Latin American | 6 |
| South Asian (East Indian, Pakistani, Sri Lankan) | 7 |
| Southeast Asian (Vietnamese, Cambodian, Laotian, Thai) | 8 |
| West Asian (Afghan, Iranian) | 9 |
| Chinese | 10 |
| Japanese | 11 |
| Korean | 12 |
| Filipino | 13 |
| Arab | 14 |
| Other, please specify _____ | 96 |
| Prefer not to say | 99 |

A8. Do you have lived experience as a member of any of the following marginalized groups? *Please select all that apply.*

| Gender-diverse (e.g., agender, non-binary, transgender, cisgender) | 1 |
| --- | --- |
| Intersex | 2 |
| LGBTQ+ (lesbian, gay, bisexual, pansexual, transgender, queer, two-spirited, questioning) | 3 |
| Indigenous | 4 |
| Racialized (i.e., person of color) | 5 |
| Neurodivergent (e.g., ADHD, autistic, dyslexic) | 6 |
| Disabled, or caregiver of a person with a disability | 7 |
| Member of a marginalized group not listed, please specify ____________ | 8 |
| None of the above | 98 |
| Prefer not to say | 99 |

A9. What is your marital status?

| Never legally married | 1 |
| --- | --- |
| Legally married | 2 |
| Separated, but still legally married | 3 |
| Divorced | 4 |
| Widowed | 5 |
| Living common law | 6 |
| Other, please specify _____ | 96 |
| Prefer not to say | 99 |

A10. How many people are currently living in your household, including yourself?

__________

A11. Which of the following categories best describes your household income, before taxes and other deductions, for the last 12 months?

| Less than $50,000 | 1 |
| --- | --- |
| $50,000 to less than $59,999 | 2 |
| $60,000 to less than $69,999 | 3 |
| $70,000 to less than $79,999 | 4 |
| $80,000 to less than $89,999 | 5 |
| $90,000 to less than $99,999 | 6 |
| $100,000 or more | 7 |
| Prefer not to say | 99 |

**SECTION B: DECISIONAL NEEDS ASSESSMENT [PN: DO NOT SHOW]**

**PN: SHOW ON A SEPARATE SCREEN**

In this section, we will ask you to share your opinion on situations in which decisions needed to be made about your health in the last year. In particular, we are interested in tough health decisions.

Definition of a tough decision:

A decision is tough when there is more than one option and there is no clear best option (see example in box 1). For this type of decision, your chosen option will depend on your personal situation and the pros and the cons of the possible options. When faced with a tough decision, people often discuss it with a health provider (e.g. doctor, nurse, social worker), family member, or a friend.

Box 1 Example of a tough decision:

Here is an example of a tough decision about the choice of treatment for knee pain caused by aging of the joints, i.e. knee arthritis. Different options to relieve knee pain may include medication, anti-inflammatory injections, surgery, or weight loss. Scientific research provides information about how well these options work to reduce pain (pros) and possible side effects (cons). You can weigh the pros and cons for each option in order to identify the option that best suits you (your preferred option). Therefore, people who have the same health problem—in this example, knee pain due to aging joints - may choose different options; one may choose to take medication while another may choose anti-inflammatory injections.

Since the COVID-19 pandemic, there are other considerations in making the decision described above (e.g., cancelled elective surgeries, delays in surgery, pain medicines, people considering alternative options, having access to people to help you at home after the surgery, access to programs such as weight loss or fitness classes) and there have been many new tough decisions.

B1. Have you made one of the following decisions during the last year? *Please select all that apply.*

**PN: RANDOMIZE**

| Decisions about COVID-19 and health care (testing for COVID-19, going to the emergency department or the doctor for COVID-19 symptoms, delaying chemotherapy, participating in clinical trials of new treatments) | 1 |
| --- | --- |
| Decisions about taking a vaccine for COVID-19 | 2 |
| Decisions about temporarily moving from a retirement home or nursing home during COVID-19 | 3 |
| Other decisions about COVID-19 such as wearing a mask, using disinfectant, limiting social contacts etc. | 4 |
| Decisions about going on a mechanical ventilator, palliative care, medical assistance in dying, advance care planning, location of care at end of life (hospital, at home, palliative care residence) | 5 |
| Decisions about managing a health condition (delaying seeking medical attention for new or unresolved symptoms, having tests, new treatments, dental visit, screening tests, admission to hospital) | 6 |
| Decisions about surgery (delaying surgery, joint replacement, prostate cancer surgery, back surgery, breast cancer surgery, etc.) | 7 |
| Decisions about taking medication (to lower cholesterol, to control blood sugar, prevent heartburn, sleeping pills, pills to control anxiety) | 8 |
| Decisions about pain management (e.g., medication, marijuana, physiotherapy) | 9 |
| Decisions about mental health treatment | 10 |
| Decisions about getting help or attending rehabilitation for an addiction or overdose | 11 |
| Decisions about pregnancy and childbirth (planning a pregnancy, prenatal testing, type/setting of childbirth, methods of feeding, unplanned pregnancy) | 12 |
| Decisions about birth control (condoms, birth control pills, vasectomy, intrauterine device (IUD), other) | 13 |
| Decisions about participating in clinical trials | 14 |
| Other decisions? please specify: __________ | 96 |

B2. Identify one specific tough or difficult health decision that you faced or are facing and are willing to focus on for the next questions. *Please select one.*

**PN: SHOW ONLY THOSE CODES SELECTED AT B1. RANDOMIZE IN THE SAME ORDER AS B1.**

**AUTO-PUNCH AND SKIP B2 IF ONLY ONE CODE SELECTED AT B1**

| Decisions about COVID-19 and health care (testing for COVID-19, going to the emergency department or the doctor for COVID-19 symptoms, delaying chemotherapy, participating in clinical trials of new treatments) | 1 |
| --- | --- |
| Decisions about taking a vaccine for COVID-19 | 2 |
| Decisions about temporarily moving from a retirement home or nursing home during COVID-19 | 3 |
| Other decisions about COVID-19 such as wearing a mask, using disinfectant, limiting social contacts etc. | 4 |
| Decisions about going on a mechanical ventilator, palliative care, medical assistance in dying, advance care planning, location of care at end of life (hospital, at home, palliative care residence) | 5 |
| Decisions about managing a health condition (delaying seeking medical attention for new or unresolved symptoms, having tests, new treatments, dental visit, screening tests, admission to hospital) | 6 |
| Decisions about surgery (delaying surgery, joint replacement, prostate cancer surgery, back surgery, breast cancer surgery, etc.) | 7 |
| Decisions about taking medication (to lower cholesterol, to control blood sugar, prevent heartburn, sleeping pills, pills to control anxiety) | 8 |
| Decisions about pain management (e.g., medication, marijuana, physiotherapy) | 9 |
| Decisions about mental health treatment | 10 |
| Decisions about getting help or attending rehabilitation for an addiction or overdose | 11 |
| Decisions about pregnancy and childbirth (planning a pregnancy, prenatal testing, type/setting of childbirth, methods of feeding, unplanned pregnancy) | 12 |
| Decisions about birth control (condoms, birth control pills, vasectomy, intrauterine device (IUD), other) | 13 |
| Decisions about participating in clinical trials | 14 |
| Other decisions? please specify: __________ **[PIPE IN FROM B1]** | 96 |

B3. What made _____ **[PN: INSERT B2 RESPONSE]** more difficult for you? *Please select all that apply.*

**PN: RANDOMIZE**

| Public health restrictions due to COVID-19 | 1 |
| --- | --- |
| Worry about getting COVID-19 | 2 |
| Worried about choosing the ‘wrong’ option | 3 |
| No or limited access to information on the decision or options | 4 |
| Difficulty in believing scientific evidence | 5 |
| Difficulty separating fake news/fake science results from real scientific evidence | 6 |
| Overloaded with information | 7 |
| Difficulty discussing the decision with health care providers | 8 |
| Difficulty discussing the decision with important others (e.g., spouse, family, friends) | 9 |
| No skills or ability for making this type of decision | 10 |
| Other, please specify _________________ | 96 |

B4. Did you make the decision **[PN: INSERT B2 RESPONSE]** alone?

| Yes | 1 |
| --- | --- |
| No | 2 |
| I have not yet made this decision | 3 |

B5. How long ago did you make this decision _____ **[PN: INSERT B2 RESPONSE]**?

| I am currently making this decision | 1 |
| --- | --- |
| I made this decision within the last month | 2 |
| I made this decision 1 to 6 months ago | 3 |
| I made this decision more than 6 months ago | 4 |
| I don’t remember | 5 |
| I prefer not to answer | 6 |

B6. What were the **options** for the difficult decision_____ **[PN: INSERT B2 RESPONSE]**? *Please specify.*

|  |
| --- |

B7a. Did you consider costs related to the options?

| Yes | 1 |
| --- | --- |
| No | 2 |

**PN: ASK IF YES SELECTED AT B7a. SHOW ON THE SAME SCREEN AFTER B7a**

B7b. To what extent did the costs affect the decision made? *Please specify.*

|  |
| --- |

B8. Which option did you **choose**? *Please specify.*

|  |
| --- |

B9. Was it your preferred option?

| Yes | 1 |
| --- | --- |
| No | 2 |

B10. The following questions concern the tough decision_____ **[PN: INSERT B2 RESPONSE]** you have made. Now think about the moment when the decision was made. Please indicate to what extent you agree or disagree with the statements by choosing which one best describes your feeling.

|  |  | Strongly Agree | Agree | Neither Agree Nor Disagree | Disagree | Strongly Disagree |
| --- | --- | --- | --- | --- | --- | --- |
| 1 | I knew which options were available to me |  |  |  |  |  |
| 2 | I knew the benefits of each option |  |  |  |  |  |
| 3 | I knew the risks and side effects of each option |  |  |  |  |  |
| 4 | I was clear about which benefits matter most to me |  |  |  |  |  |
| 5 | I was clear about which risks and side effects matter most to me |  |  |  |  |  |
| 6 | I was clear about which was more important to me (the benefits or the risks and side effects) |  |  |  |  |  |
| 7 | I had enough support from others to make a choice |  |  |  |  |  |
| 8 | I chose without pressure from others |  |  |  |  |  |
| 9 | I had enough advice to make a choice |  |  |  |  |  |
| 10 | I was clear about the best choice for me |  |  |  |  |  |
| 11 | I felt sure about what to choose |  |  |  |  |  |
| 12 | This decision was easy for me to make |  |  |  |  |  |
| 13 | I felt I had made an informed choice |  |  |  |  |  |
| 14 | My decision showed what is important for me |  |  |  |  |  |
| 15 | I expect to stick with my decision |  |  |  |  |  |
| 16 | I am satisfied with my decision |  |  |  |  |  |

B11. During the decision-making process for the tough decision _____ **[PN: INSERT B2 RESPONSE]**, please indicate how you reached your decision:

| I made the decision alone | 1 |
| --- | --- |
| I made the decision alone but considered the opinion of my health care provider | 2 |
| My health care provider and I decided together, equally | 3 |
| My health care provider made the decision but considered my opinion | 4 |
| My health care provider made the decision alone | 5 |

B12. If you had to do it all over again, how would you prefer the decision be made?

| I would make the decision alone | 1 |
| --- | --- |
| I would make the decision alone but consider the opinion of my health care provider | 2 |
| My health care provider and I would decide together, equally | 3 |
| My health care provider would make the decision but consider my opinion | 4 |
| My health care provider would make the decision alone | 5 |

B13. Did you feel you had the ability or skill to make this type of decision?

| Yes | 1 |
| --- | --- |
| No | 2 |
| Unsure | 3 |

B14. What sources of information would you consider trustworthy? *Please select all that apply.*

**PN: RANDOMIZE**

| Organizations interested in these kinds of difficult decisions (e.g. pharmacies, Canadian Arthritis Society, Canadian Cancer Society) | 1 |
| --- | --- |
| Health professional (e.g. doctor, nurse, social worker) | 2 |
| Health Canada | 3 |
| Provincial health departments | 4 |
| Health insurance companies | 5 |
| Consumer or patient associations | 6 |
| Companies that produce health information | 7 |
| Other, please specify: __________ | 96 |

B15. Now please think about the tough decision _____ **[PN: INSERT B2 RESPONSE]** that was made. Indicate whether you agree or disagree with the following statements by choosing which one best represents your opinion about this decision.

|  |  | Strongly Agree | Agree | Neither Agree Nor Disagree | Disagree | Strongly Disagree |
| --- | --- | --- | --- | --- | --- | --- |
| 1 | It was the right decision |  |  |  |  |  |
| 2 | I regret the choice that was made |  |  |  |  |  |
| 3 | I would go for the same choice if I had to do it over again |  |  |  |  |  |
| 4 | The choice did me a lot of harm |  |  |  |  |  |
| 5 | The decision was a wise one |  |  |  |  |  |

B16. Taking everything in **YOUR** life into account, please rate **your** overall Quality of life (QoL) on the following 7 points scale.

One (1) means life is very distressing; it’s hard to imagine how it could get much worse.

Seven (7) means life is great; it’s hard to imagine how it could get much better.

Four (4) means life is so-so, neither good nor bad.

Now where are you? Select a number on the figure below that best describes your current overall QoL.

| 1 | 2 | 3 | 4 | 5 | 6 | 7 |
| --- | --- | --- | --- | --- | --- | --- |
| Life is very distressing |  |  | Life is so-so |  |  | Life is great |

**NEXT SCREEN:**

This concludes our survey. Thank you very much for your participation.

**SUPPORTING CANADIANS MAKING HEALTH DECISIONS: A DECISIONAL NEEDS ASSESSMENT DURING THE COVID-19 PANDEMIC – QUESTIONNAIRE**

**(FRENCH VERSION)**

**SCREENER**

S.0 Would you prefer to complete the survey in English or French?

Préféreriez-vous répondre à ce questionnaire en anglais ou en français?

*Please select one answer only.*

*Veuillez sélectionner une seule réponse.*

| a. | 🔾 | English / Anglais | Go to S1 in English |
| --- | --- | --- | --- |
| b. | 🔾 | French / Français | Go to S1 in French |

S1. Quelle est votre date de naissance? *Veuillez inscrire le jour/le mois/l’année.*

**PN: PLEASE PROGRAM A CALENDAR**

**___________**

**PN: THANK AND TERMINATE IF LESS THAN 18 YEARS OLD**

S2**.** Avez-vous reçu des services de santé au cours des 12 derniers mois?

| Oui | 1 |  |
| --- | --- | --- |
| Non | 2 | **THANK AND TERMINATE** |

S3**.** Quelle est la date (mois et année) de la dernière fois où vous avez reçu des services de santé? *Veuillez inscrire le jour/le mois/l’année.*

**___________**

**PN: THANK AND TERMINATE IF MORE THAN 12 MONTHS AGO**

**PN: SHOW ON A SEPARATE SCREEN**

Vous êtes invité(e) à participer à un sondage qui s’adresse aux adultes recevant actuellement ou ayant reçu des services de santé au cours de la dernière année. Cette étude vise à mieux comprendre les besoins des adultes au moment de la prise de décisions concernant leur santé. Le sondage dure environ 15 à 20 minutes. Ce projet de recherche est financé par les Instituts de recherche en santé du Canada. Pour de plus amples informations sur cette étude, veuillez communiquer par courriel avec Meg Carley, Responsable du projet de recherche ou consultez le site web de la recherche : <https://decisionaid.ohri.ca/francais/>.

**MAIN QUESTIONNAIRE**

**SECTION A: SOCIO-DEMOGRAPHIC INFORMATION ON PARTICIPANTS [PN: DO NOT SHOW]**

**PN: SHOW ON A SEPARATE SCREEN**

Pour commencer le sondage, nous aimerions tout d’abord vous poser quelques questions générales sur vous. Ces renseignements nous aideront à décrire le groupe de personnes qui ont répondu au sondage et à analyser les résultats.

A1. Quelle est votre province/territoire de résidence?

| Terre-Neuve | 1 |
| --- | --- |
| Île-du-Prince-Édouard | 2 |
| Nouvelle-Écosse | 3 |
| Nouveau-Brunswick | 4 |
| Québec | 5 |
| Ontario | 6 |
| Manitoba | 7 |
| Saskatchewan | 8 |
| Alberta | 9 |
| Colombie-Britannique | 10 |
| Nunavut | 11 |
| Territoires du Nord-Ouest | 12 |
| Yukon | 13 |
| Autre, svp spécifiez _____ | 96 |

A2. Quel était votre sexe à la naissance? Par sexe, on entend le sexe assigné à la naissance.

| Masculin | 1 |
| --- | --- |
| Féminin | 2 |
| Préfère ne pas répondre | 99 |

A3. Quel est votre genre? Par genre, on entend le genre actuel, qui peut différer du sexe assigné à la naissance ou de celui inscrit dans les documents légaux.

| Masculin | 1 |
| --- | --- |
| Féminin | 2 |
| Autre, svp spécifiez _____ | 96 |
| Préfère ne pas répondre | 99 |

A4. Quelle est la première langue que vous avez apprise à la maison dans votre enfance et que vous comprenez encore?

| Français | 1 |
| --- | --- |
| Anglais | 2 |
| Une langue autochtone | 3 |
| Espagnol | 4 |
| Mandarin | 5 |
| Arabe | 6 |
| Autre, svp spécifiez _____ | 96 |

A5. Quel est le plus haut niveau de scolarité que vous ayez atteint?

| Niveau inférieur à un diplôme d’études secondaires ou à son équivalent | 1 |
| --- | --- |
| Diplôme d’études secondaires ou un certificat d’équivalence d’études secondaires | 2 |
| Certificat ou diplôme d’un collège, d’un cégep ou d’un autre établissement non universitaire (autre: certificats ou diplômes de métiers) | 3 |
| Certificat ou diplôme universitaire inférieur au baccalauréat | 4 |
| Baccalauréat (p. ex. B.A., B.Sc., LL.B.) | 5 |
| Certificat, diplôme ou grade universitaire supérieur au baccalauréat | 6 |
| Autre, svp spécifiez _____ | 96 |
| Préfère ne pas répondre | 99 |

A6. Veuillez indiquer les 3 premiers caractères de votre code postal.

__________ **[PN: ALLOW 3 DIGITS]**

A7. Les gens qui habitent au Canada ont des origines culturelles et ethniques très variées. Êtes-vous...?

| Membre des Premières Nations | 1 |
| --- | --- |
| Métis | 2 |
| Inuk | 3 |
| Blanc | 4 |
| Noir | 5 |
| Latino-Américain | 6 |
| Sud-Asiatique (Indien de l’Inde, Pakistanais, Sri-Lankais) | 7 |
| Asiatique du Sud-Est (Vietnamien, Cambodgien, Laotien, Thaïlandais) | 8 |
| Asiatique occidental (Afghan, Iranien) | 9 |
| Chinois | 10 |
| Japonais | 11 |
| Coréen | 12 |
| Philippin | 13 |
| Arabe | 14 |
| Autre, svp spécifiez _____ | 96 |
| Préfère ne pas répondre | 99 |

A8. Avez-vous vécu une expérience en tant que membre de l'un des groupes marginalisés suivants? *Veuillez sélectionner toutes les réponses qui s’appliquent.*

| Diversité de genre (par exemple, agenre, non binaire, transgenre, cisgenre) | 1 |
| --- | --- |
| Intersexe | 2 |
| LGBTQ+ (lesbienne, gai, bisexuel, pansexuel, transgenre, queer, bispirituel, en questionnement) | 3 |
| Indigène | 4 |
| Racialisé (c'est-à-dire personne de couleur) | 5 |
| Neurodivergent (par exemple, TDAH, autiste, dyslexique) | 6 |
| Handicapé, ou soignant d'une personne handicapée | 7 |
| Membre d'un groupe marginalisé non répertorié, veuillez préciser _____ | 8 |
| Aucune des réponses ci-dessus | 98 |
| Préfère ne pas répondre | 99 |

A9. Quel est votre état matrimonial?

| Jamais légalement marié | 1 |
| --- | --- |
| Légalement marié (et non séparé) | 2 |
| Séparé, mais toujours légalement marié | 3 |
| Divorcé | 4 |
| Veuf ou veuve | 5 |
| En union libre | 6 |
| Autre, svp spécifiez _____ | 96 |
| Préfère ne pas répondre | 99 |

A10. Combien de personnes vivent actuellement dans votre ménage, y compris vous-même?

__________

A11. Laquelle de ces catégories décrit le mieux votre revenu familial combiné, avant impôts et autres déductions, pour les 12 derniers mois?

| Moins de 50 000 $ | 1 |
| --- | --- |
| Entre 50 000 $ et moins de 59 999 $ | 2 |
| Entre 60 000 $ et moins de 69 999 $ | 3 |
| Entre 70 000 $ et moins de 79 999 $ | 4 |
| Entre 80 000 $ et moins de 89 999 $ | 5 |
| Entre 90 000 $ et moins de 99 999 $ | 6 |
| 100 000 $ et plus | 7 |
| Préfère ne pas répondre | 99 |

**SECTION B: DECISIONAL NEEDS ASSESSMENT [PN: DO NOT SHOW]**

**PN: SHOW ON A SEPARATE SCREEN**

Dans cette section, nous allons vous demander de nous faire part de votre opinion sur les situations au sein desquelles il est nécessaire de prendre des décisions qui sont en lien avec votre santé au cours de la dernière année. Tout particulièrement, nous nous intéressons aux décisions plus difficiles en matière de santé.

Définition d’une décision difficile :

Une décision est difficile lorsqu’il y a plus d’une option et qu’il n’y a pas, à priori, d’option qui soit clairement meilleure (voir l’encadré 1). Pour ce type de décision, votre choix dépendra de votre situation personnelle et le pour et le contre de chacune des options possibles. Face à une décision difficile, les gens en discutent souvent avec un professionnel de la santé (ex. médecin, infirmière, travailleur social), les membres de leur famille ou leurs amis.

Encadré 1 Exemple d’une décision difficile :

Voici un exemple de décision difficile sur le choix de traitement à suivre lorsqu’on a une douleur au genou causée par le vieillissement des articulations (c.-à-d. arthrose du genou). Différentes options peuvent être considérées pour soulager la douleur aux genoux. Celles-ci peuvent inclure : médicaments, injections anti-inflammatoires, chirurgie ou perte de poids. Pour chacune de ces options, les recherches scientifiques nous informent sur leur efficacité respective à soulager la douleur (avantage) et les possibles effets secondaires (inconvénient). Seul vous pourrez peser le pour et le contre de chacune des options afin de bien cerner le choix qui vous convient le mieux. Ainsi, des personnes qui ont le même problème de santé, dans l’exemple présent une douleur au genou due au vieillissement des articulations, pourraient faire des choix différents : le premier pourrait choisir le médicament, alors que l’autre opterait pour l’injection anti-inflammatoire.

Depuis le début de la pandémie de COVID-19, d’autres facteurs entrent en considération dans la prise de décision décrite précédemment (annulation des opérations non urgentes, chirurgies retardées, médicaments contre la douleur, solutions de rechange envisagées par les gens, possibilité d’avoir de l’aide à la maison après la chirurgie, accès à des programmes comme des cours d’activité physique ou de perte de poids) et les gens ont dû prendre de nombreuses nouvelles décisions difficiles.

B1. Au cours de la dernière année, avez-vous dû prendre les décisions suivantes? *Veuillez sélectionner toutes les réponses qui s’appliquent.*

**PN: RANDOMIZE**

| Décisions à propos de la COVID-19 et des soins de santé (test de dépistage de la COVID-19, se rendre à l’urgence ou chez le médecin en raison de symptômes de la COVID-19, reporter des séances de chimiothérapie, participer à des essais cliniques pour de nouveaux traitements). | 1 |
| --- | --- |
| Décisions à propos d’un vaccin contre la COVID-19. | 2 |
| Décisions à propos d’un déménagement temporaire hors d’un établissement de soins de longue durée (maison de retraite, foyer de soins infirmiers) pendant la COVID-19. | 3 |
| Autres décisions à propos de la COVID-19 (port du masque, utilisation de désinfectant, réduction des contacts sociaux, etc.). | 4 |
| Décisions concernant la ventilation artificielle, les soins palliatifs, l’aide médicale à mourir, la planification préalable des soins, le choix du lieu de soins en fin de vie (hôpital, domicile, résidence de soins palliatifs). | 5 |
| Décisions à propos de la gestion d’une condition médicale (attendre avant de consulter pour de nouveaux symptômes ou des symptômes non réglés, se faire tester, commencer de nouveaux traitements, aller chez le dentiste, passer un test de dépistage, être admis à l’hôpital). | 6 |
| Décisions à propos d’une chirurgie (report, remplacement d’une articulation, chirurgie pour le cancer de la prostate, chirurgie au dos, chirurgie pour le cancer du sein, etc.). | 7 |
| Décisions à propos de la prise de médicament (pour diminuer le cholestérol, contrôler la glycémie ou prévenir les brûlures d’estomac, somnifères, pilules pour contrôler l’anxiété). | 8 |
| Décisions à propos de la gestion de la douleur (médication, marijuana, physiothérapie). | 9 |
| Décisions à propos de traitements en santé mentale. | 10 |
| Décisions à propos d’aide ou de traitement concernant une dépendance ou une surdose. | 11 |
| Décisions à propos d’une grossesse ou d’un accouchement (planification d’une grossesse, tests prénataux, type/contexte d’accouchement, méthodes d’allaitement, grossesse imprévue). | 12 |
| Décisions à propos de la contraception (condom, pilule contraceptive, vasectomie, dispositif intra-utérin, autre). | 13 |
| Décisions à propos de la participation à des essais cliniques. | 14 |
| Autre(s) décision(s), svp spécifiez: __________ | 96 |

B2. Veuillez identifier une décision de santé particulièrement difficile que vous avez dû ou que vous devez prendre et sur laquelle vous acceptez de répondre dans les questions suivantes. *Veuillez sélectionner une seule réponse.*

**PN: SHOW ONLY THOSE CODES SELECTED AT B1. RANDOMIZE IN THE SAME ORDER AS B1.**

**AUTO-PUNCH AND SKIP B2 IF ONLY ONE CODE SELECTED AT B1**

| Décisions à propos de la COVID-19 et des soins de santé (test de dépistage de la COVID-19, se rendre à l’urgence ou chez le médecin en raison de symptômes de la COVID-19, reporter des séances de chimiothérapie, participer à des essais cliniques pour de nouveaux traitements). | 1 |
| --- | --- |
| Décisions à propos d’un vaccin contre la COVID-19. | 2 |
| Décisions à propos d’un déménagement temporaire hors d’un établissement de soins de longue durée (maison de retraite, foyer de soins infirmiers) pendant la COVID-19. | 3 |
| Autres décisions à propos de la COVID-19 (port du masque, utilisation de désinfectant, réduction des contacts sociaux, etc.). | 4 |
| Décisions concernant la ventilation artificielle, les soins palliatifs, l’aide médicale à mourir, la planification préalable des soins, le choix du lieu de soins en fin de vie (hôpital, domicile, résidence de soins palliatifs). | 5 |
| Décisions à propos de la gestion d’une condition médicale (attendre avant de consulter pour de nouveaux symptômes ou des symptômes non réglés, se faire tester, commencer de nouveaux traitements, aller chez le dentiste, passer un test de dépistage, être admis à l’hôpital). | 6 |
| Décisions à propos d’une chirurgie (report, remplacement d’une articulation, chirurgie pour le cancer de la prostate, chirurgie au dos, chirurgie pour le cancer du sein, etc.). | 7 |
| Décisions à propos de la prise de médicament (pour diminuer le cholestérol, contrôler la glycémie ou prévenir les brûlures d’estomac, somnifères, pilules pour contrôler l’anxiété). | 8 |
| Décisions à propos de la gestion de la douleur (médication, marijuana, physiothérapie). | 9 |
| Décisions à propos de traitements en santé mentale. | 10 |
| Décisions à propos d’aide ou de traitement concernant une dépendance ou une surdose. | 11 |
| Décisions à propos d’une grossesse ou d’un accouchement (planification d’une grossesse, tests prénataux, type/contexte d’accouchement, méthodes d’allaitement, grossesse imprévue). | 12 |
| Décisions à propos de la contraception (condom, pilule contraceptive, vasectomie, dispositif intra-utérin, autre). | 13 |
| Décisions à propos de la participation à des essais cliniques. | 14 |
| Autre décision, svp spécifiez: __________ **[PIPE-IN FROM B1]** | 96 |

B3. Qu’est-ce qui a rendu cette _____ **[PN: INSERT B2 RESPONSE]** plus difficile? *Veuillez sélectionner toutes les réponses qui s’appliquent.*

**PN: RANDOMIZE**

| Les restrictions de la santé publique en raison de la COVID-19 | 1 |
| --- | --- |
| Crainte d’attraper la COVID-19 | 2 |
| Crainte de faire le « mauvais choix » | 3 |
| Pas ou peu d’accès à de l’information sur la décision ou les choix | 4 |
| Difficulté à faire confiance à la science | 5 |
| Difficulté à faire la différence entre les fausses nouvelles ou les faux résultats scientifiques et les vraies données scientifiques | 6 |
| Surcharge d’information | 7 |
| Difficulté à discuter de la décision avec des professionnels de la santé | 8 |
| Difficulté à discuter de la décision avec d’autres personnes importantes (conjoint(e), famille, amis) | 9 |
| Absence d’aptitude ou de compétence à prendre ce type de décision | 10 |
| Autre, svp spécifiez _____ | 96 |

B4. Avez-vous pris cette décision seul(e) **[PN: INSERT B2 RESPONSE]**?

| Oui | 1 |
| --- | --- |
| Non | 2 |
| Je n’ai pas encore pris ma décision | 3 |

B5. Cela fait combien de temps que vous avez pris cette décision _____ **[PN: INSERT B2 RESPONSE]**?

| Je suis en processus de décision | 1 |
| --- | --- |
| J’ai pris ma décision il y a moins d’un mois | 2 |
| J’ai pris ma décision il y a entre un et six mois | 3 |
| J’ai pris ma décision il y a plus de six mois | 4 |
| Je ne m’en souviens pas | 5 |
| Je préfère ne pas répondre | 6 |

B6. Quelles étaient les **options** que vous avez eu à envisager pour cette difficile décision ____**[PN: INSERT B2 RESPONSE]**? Svp spécifiez

|  |
| --- |

B7a. Avez-vous tenu compte des coûts rattachés à chaque option?

| Oui | 1 |
| --- | --- |
| Non | 2 |

**PN: ASK IF YES SELECTED AT B7a. SHOW ON THE SAME SCREEN AFTER B7a**

B7b. Si oui, quel poids ce facteur a-t-il eu dans votre décision? Svp spécifiez :

|  |
| --- |

B8. Quelle option avez-vous **choisie**? Svp spécifiez :

|  |
| --- |

B9. Était-ce votre option préférée?

| Oui | 1 |
| --- | --- |
| Non | 2 |

B10. Les questions suivantes portent sur la difficile décision _____ **[PN: INSERT B2 RESPONSE]** que vous avez prise. Pensez maintenant au moment où vous avez pris votre décision et veuillez nous indiquer dans quelle mesure vous êtes en accord ou en désaccord avec ces énoncés en choisissant ce qui décrit le mieux votre sentiment.

|  |  | Tout à fait d’accord | D’accord | Ni d’accord ni pas d’accord | Pas d’accord | Pas de tout d’accord |
| --- | --- | --- | --- | --- | --- | --- |
| 1 | Je connaissais les options qui s’offraient à moi |  |  |  |  |  |
| 2 | Je connaissais les bénéfices de chaque option |  |  |  |  |  |
| 3 | Je connaissais les risques et les effets secondaires de chaque option |  |  |  |  |  |
| 4 | J’étais certain des bénéfices les plus importants pour moi |  |  |  |  |  |
| 5 | J’étais certain des risques et effets secondaires qui importent le plus pour moi |  |  |  |  |  |
| 6 | J’étais certain de ce qui est le plus important pour moi (les bénéfices ou les risques et effets secondaires) |  |  |  |  |  |
| 7 | J’avais suffisamment de soutien des autres pour faire un choix |  |  |  |  |  |
| 8 | J’ai fait mon choix sans pression des autres |  |  |  |  |  |
| 9 | J’avais suffisamment de conseils pour faire un choix |  |  |  |  |  |
| 10 | J’étais certain du meilleur choix pour moi |  |  |  |  |  |
| 11 | J’étais certain du choix à faire |  |  |  |  |  |
| 12 | Il a été facile pour moi de prendre la décision |  |  |  |  |  |
| 13 | J’avais le sentiment d’avoir fait un choix éclairé |  |  |  |  |  |
| 14 | Ma décision mettait en évidence ce qui est important pour moi |  |  |  |  |  |
| 15 | Je compte maintenir ma décision |  |  |  |  |  |
| 16 | Je suis satisfait de ma décision |  |  |  |  |  |

B11. Au cours de la prise de cette difficile décision _____ **[PN: INSERT B2 RESPONSE]**, veuillez indiquer comment celle-ci a été prise :

| J’ai pris la décision seul(e) | 1 |
| --- | --- |
| J’ai pris la décision seul(e), mais en considérant l’opinion de mon professionnel de santé | 2 |
| Mon professionnel de santé et moi avons décidé ensemble | 3 |
| Mon professionnel de santé a pris la décision, mais en considérant mon opinion | 4 |
| Mon professionnel de santé a pris la décision seul | 5 |

B12. Si vous deviez recommencer, de quelle manière préféreriez-vous prendre la décision?

| Je prendrais la décision seul(e) | 1 |
| --- | --- |
| Je prendrais la décision seul(e), mais en considérant l’opinion de mon professionnel de santé | 2 |
| Mon professionnel de santé et moi déciderions ensemble | 3 |
| Mon professionnel de santé prendrait la décision, mais en considérant mon opinion | 4 |
| Mon professionnel de santé prendrait la décision seul | 5 |

B13. Avez-vous eu l’impression de posséder l’aptitude ou la compétence requise pour prendre ce type de décision?

| Oui | 1 |
| --- | --- |
| Non | 2 |
| Pas sûr | 3 |

B14. Parmi la liste suivante, quelles sources d’information considérez-vous comme fiables? *Veuillez sélectionner toutes les réponses qui s’appliquent.*

**PN: RANDOMIZE**

| Organisations s’intéressant à ce genre de décision difficile (ex. pharmacies, Société canadienne d’arthrite, Société canadienne du cancer) | 1 |
| --- | --- |
| Professionnels de la santé (ex. médecin, infirmière, travailleur social) | 2 |
| Santé Canada | 3 |
| Ministères de la Santé provinciaux | 4 |
| Compagnies d’assurance maladie | 5 |
| Associations de consommateurs ou de patients | 6 |
| Compagnies qui produisent des informations sur la santé | 7 |
| Autres, svp spécifiez : | 96 |

B15. Maintenant, pensez à la difficile décision _____ **[PN: INSERT B2 RESPONSE]** que vous avez prise. Indiquez si vous êtes en accord ou en désaccord avec les énoncés suivants en choisissant ce qui correspond le mieux à votre opinion concernant cette décision.

|  |  | Fortement en accord | Plutôt en accord | Ni en accord ni en désaccord | Plutôt en désaccord | Fortement en désaccord |
| --- | --- | --- | --- | --- | --- | --- |
| 1 | C’était la bonne décision |  |  |  |  |  |
| 2 | Je regrette le choix qui a été fait |  |  |  |  |  |
| 3 | Je referai le même choix si j’avais à le faire à nouveau |  |  |  |  |  |
| 4 | Ce choix m’a causé beaucoup de tort |  |  |  |  |  |
| 5 | C’était une sage décision |  |  |  |  |  |

B16. En considérant tout ce qui se passe dans votre vie, veuillez évaluer votre qualité de vie globale sur l’échelle à 7 points suivante.

Un (1) signifie que la vie est très pénible; c’est difficile d’imaginer comment cela pourrait être pire.

Sept (7) signifie que la vie est excellente; c’est difficile d’imaginer comment cela pourrait aller mieux.

Quatre (4) signifie que la vie est juste correcte, ni bonne ni mauvaise.

Maintenant, où vous situez-vous? Cliquez sur le chiffre de l’échelle ci-dessous qui décrit le mieux votre qualité de vie globale courante.

| 1 | 2 | 3 | 4 | 5 | 6 | 7 |
| --- | --- | --- | --- | --- | --- | --- |
| La vie est très pénible |  |  | La vie est juste correcte |  |  | La vie est excellente |

**NEXT SCREEN:**

Ceci conclut notre sondage. Merci beaucoup de votre participation.
