# Supplementary material for: Decisions and Decisional Needs of Canadians From all Provinces and Territories During the COVID-19 Pandemic: Population-Based Cross-sectional Surveys
Source: JMIR Public Health Surveill. 2023 Mar 21;9:e43652. doi: 10.2196/43652 (PMC10131685; doi:10.2196/43652)
Supplement: Multimedia Appendix 2 [file publichealth_v9i1e43652_app2.docx]

Appendix 2: Comparison of respondents to those invited and the Canadian census

|  | Invites | Respondents | Census |
| --- | --- | --- | --- |
| **Gender** | | | |
| Males | 52.7% | 46.1% | 49.3% |
| Females | 47.3% | 53.1% | 50.7% |
| **Province** | | | |
| AB | 11.7% | 12.2% | 11.6% |
| BC | 13.8% | 16.4% | 13.5% |
| SK | 3.4% | 2.9% | 3.1% |
| MB | 3.3% | 3.3% | 3.6% |
| ON | 37.9% | 38.6% | 38.6% |
| QC | 21.1% | 18.3% | 22.6% |
| NB | 2.8% | 2.5% | 2.1% |
| NF | 1.5% | 1.8% | 1.4% |
| NS | 2.8% | 3.1% | 2.6% |
| PE | 0.8% | 0.6% | 0.4% |
| NT | 0.2% | 0% | 0.1% |
| NU | 0.2% | 0% | 0.1% |
| YT | 0.4% | 0.5% | 0.1% |
| **Age** | | | |
| 18-24 | 22.3% | 9.0% | 10.1% |
| 25-34 | 21.8% | 14.4% | 16.6% |
| 35-44 | 18.4% | 16.1% | 16.5% |
| 45-54 | 17.5% | 20.1% | 15.7% |
| 55-64 | 10.0% | 15.3% | 17.5% |
| 65-74 | 7.1% | 17.9% | 13.6% |
| 75+ | 2.9% | 7.2% | 10.0% |
| **Education** | | | |
| High school or less | 33.7% | 20.5% | 44.8% |
| College, CEGEP or other non-university certificate or diploma (other: trades certificates or diplomas) | 26.3% | 30.2% | 29.4% |
| University certificate or diploma below bachelor level | 9.2% | 6.1% | 2.8% |
| University (Bachelor’s or higher) | 30.7% | 42% | 23.2% |
